# Resistance to BmNPV via Overexpression of an Exogenous Gene Controlled by an Inducible Promoter and Enhancer in Transgenic Silkworm, *Bombyx mori*

**DOI:** 10.1371/journal.pone.0041838

**Published:** 2012-08-01

**Authors:** Liang Jiang, Tingcai Cheng, Ping Zhao, Qiong Yang, Genhong Wang, Shengkai Jin, Ping Lin, Yang Xiao, Qingyou Xia

**Affiliations:** 1 State Key Laboratory of Silkworm Genome Biology, Southwest University, Chongqing, P. R. China; 2 Sericulture and Farm Product Processing Research Institute, Guangdong Academy of Agricultural Sciences, Guangzhou, China; National University of Singapore, Singapore

## Abstract

The *hycu-ep32* gene of *Hyphantria cunea* NPV can inhibit *Bombyx mori* nucleopolyhedrovirus (BmNPV) multiplication in co-infected cells, but it is not known whether the overexpression of the *hycu-ep32* gene has an antiviral effect in the silkworm, *Bombyx mori*. Thus, we constructed four transgenic vectors, which were under the control of the 39 K promoter of BmNPV (39 KP), *Bombyx mori* A4 promoter (A4P), hr3 enhancer of BmNPV combined with 39 KP, and hr3 combined with A4P. Transgenic lines were created via embryo microinjection using practical diapause silkworm. qPCR revealed that the expression level of *hycu-ep32* could be induced effectively after BmNPV infection in transgenic lines where *hycu-ep32* was controlled by hr3 combined with 39 KP (i.e., HEKG). After oral inoculation of BmNPV with 3 × 10^5^ occlusion bodies per third instar, the mortality with HEKG-B was approximately 30% lower compared with the non-transgenic line. The economic characteristics of the transgenic lines remained unchanged. These results suggest that overexpression of an exogenous antiviral gene controlled by an inducible promoter and enhancer is a feasible method for breeding silkworms with a high antiviral capacity.

## Introduction

*Bombyx mori* nucleopolyhedrovirus (BmNPV) is a member of the Baculoviridae family. Two virion phenotypes occur during the NPV infection cycle, i.e., occlusion-derived virus (ODV) and budded virus (BV). ODV causes contagion among individual silkworms (*Bombyx mori*), whereas BV induces systemic infection throughout the host body [1], [2]. In natural conditions, NPV infects host larvae mainly via the oral route by ODV. After the ODV nucleocapsids have invaded the host cells, the viral genes begin transcription in a temporal manner to initiate a primary infection [3]. The expression of the immediate early genes of *Autographa californica* multiplenucleopolyhedrovirus (AcMNPV), such as *ie-1* and *ie-2*, begins within the first 6 hours post-infection (hpi) [4]. DNA replication can be detected initially at 6–8 hpi, before peaking at approximately 18 hpi [5]. The BV virions are produced next, which cause secondary infection at approximately 20 hpi [4]. The BmNPV life cycle is a slightly slower than that of AcMNPV. BmNPV is a primary pathogen of silkworms, which causes severe economic losses to sericulture each year. However, no effective strategy exists for controlling the virus.

Disease-resistant species can be constructed using transgenic technology. Various methods have been used to create disease-resistant species, such as knocking down the pathogenic genes via transgenic RNAi [6], [7], and overexpressing the endogenous anti-pathogen genes [8], [9], [10] or exogenous resistant genes [11], [12]. Several methods have been proposed to ensure the effectiveness of anti-disease factors and their high level expression in transgenic hosts, including the use of a constitutive promoter, an inducible promoter, a constitutive promoter combined with an enhancer, or an inducible promoter combined with an enhancer. Previous studies have reported the control of anti-disease factors by constitutive promoters [8], [9], [11], [12], which suggests that anti-disease factors could be expressed in a sustained manner, regardless of whether an infection is caused by a pathogen. However, a better method might be the increased expression of an anti-pathogen factor with an increase in the pathogen content after infection in the transgenic host, although there are no reports on this subject.

An ideal method of breeding antiviral silkworm lines would be the overexpression of an exogenous antiviral gene under the control of an inducible promoter and enhancer in the presence of the virus in transgenic silkworm. Baculovirus genomes contain homologous regions (hrs) formed of repeated sequences [13], [14]. The hrs site is the origin of viral DNA replication [15], [16] and certain hrs sits can enhance the activity of NPV early promoters, such as 39 K [13], [14], [17], p35 [18], [19], and ie-N [20]. Some hrs can also act as enhancers of non-viral promoters, such as the *B. mori* cytoplasmic actin3 gene (A3) promoter [21] and *Drosophila* hsp70 promoter [22]. The 39 K gene of AcMNPV is a delayed early gene with no transcriptional activity in uninfected cells, but it can be activated during co-transfection with a virus [17]. In the presence of an hrs upstream of the 39 K promoter, the activity of 39 K is enhanced significantly during transactivation with IE1 protein [13], [23]. The IE1 protein is the primary transcriptional regulator of NPV [24], [25], which increases the promoter activity significantly by binding to the palindromic 28 bp repeats of hrs [25], [26], [27], [28], [29].

If BmNPV and *Hyphantria cunea* NPV (HycuNPV) are co-infected in BmN-4 cells, the *hycu-ep32* gene of HycuNPV induces a global protein synthesis shutdown that inhibits BmNPV proliferation [30]. Shirata et al. [30] found that *hycu-ep32* is an early and nonessential gene that encodes a polypeptide containing 312 amino acids, with no characteristic motifs or domains. A homologue of *hycu-ep32* gene is present in the *Orgyia pseudotsugata* multicapsid NPV (OpMNPV), but it is not known in any other organism [30]. To the best of our knowledge, this is the only report of an exogenous gene that can inhibit BmNPV proliferation.

In the current study, we selected the exogenous antiviral gene *hycu-ep32* and we used the inducible 39 K promoter of BmNPV (39 KP) and the hr3 enhancer of BmNPV to construct a transgenic overexpression vector where *hycu-ep32* was controlled by hr3 combined with 39 KP. The embryos of practical diapause silkworm strain “932” were used for transgenic microinjection. Exogenous *hycu-ep32* was expressed successfully in the transgenic lines. The induced mRNA expression level of *hycu-ep32* was significantly increased in transgenic lines after infection with BmNPV. Compared with the non-transgenic line, the transgenic lines had significantly enhanced resistance and unaffected economic characteristics. This is the first report of the overexpression of an exogenous gene to enhance the antiviral ability of a practical silkworm strain using an inducible promoter and enhancer.

## Results

### Overexpression Vector Construction and Screening of Transgenic Silkworm Lines

**Figure 1:**
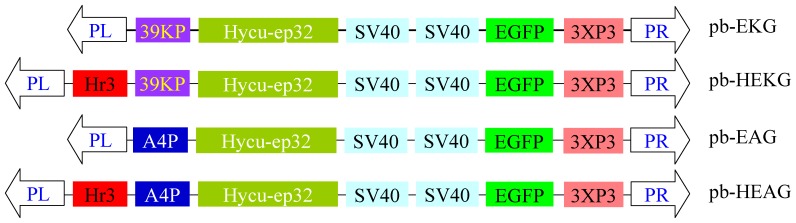
Schematic diagram of the transgenic overexpression vectors. *PiggyBac* [3×p3 EGFP afm] was a basic transgenic vector with a report marker 3×P3-EGFP-SV40. PL and PR indicate the left and right terminal inverted repeats, respectively. 39 KP and A4P represent the 39 K promoter of BmNPV and A4 promoter of *B. mori*, respectively. Hr3 is an enhancer from BmNPV. Hycu-ep32 typifies the coding sequences of the *hycu-ep32* gene, while SV40 indicates the polyadenylation signal.

The nucleotide sequences of *hycu-ep32* and 39 KP were cloned from the genomic DNA of HycuNPV and BmNPV, respectively. The overexpression vectors pb-EKG, pb-HEKG, pb-EAG, and pb-HEAG (Fig. 1) were constructed using the transgenic plasmid *piggyBac* [3×p3 EGFP afm], where *hycu-ep32* was driven by 39 KP, 39 KP combined with hr3, A4 promoter (A4P), and A4P combined with hr3, respectively. Microinjection was performed using mixtures of the overexpression plasmid and helper vector pHA3PIG [10], [31], [32] in 932 nondiapause embryos. Table 1 shows the transformation results of the embryo microinjection. G0 moths were mated with each other or backcrossed to produce G1 offspring. The G1 broods were screened for EGFP-positive expression and transgenic G1 moths in the same broods were sibling-mated to generate offspring [10]. Finally, we obtained a transgenic line from each of the vectors pb-EKG, pb-EAG, and pb-HEAG, and we named the corresponding transgenic lines as EKG, EAG, and HEAG, respectively. Eight transgenic lines of pb-HEKG were obtained and two lines (HEKG-A and HEKG-B) were selected randomly for subsequent detection experiments. Each transgenic line was sibling-mated to generate offspring for each generation.

**Table 1:** The results of embryo microinjection.

Injectedvector	Injectedstrain	Injectedembryos	Hatchedlarvae	Efficiencyof hatching	G1 broods	Broods with EGFPpositive	Efficiency ofpositive
pb-EKG	932	91	25	27.47%	6	1	16.67%
pb-EAG	932	67	18	26.87%	5	1	20.00%
pb-HEKG	932	183	47	25.68%	13	8	61.54%
pb-HEAG	932	51	11	21.57%	2	1	50.00%

### Analysis of Insertion Sites in Transgenic Lines

**Figure 2:**
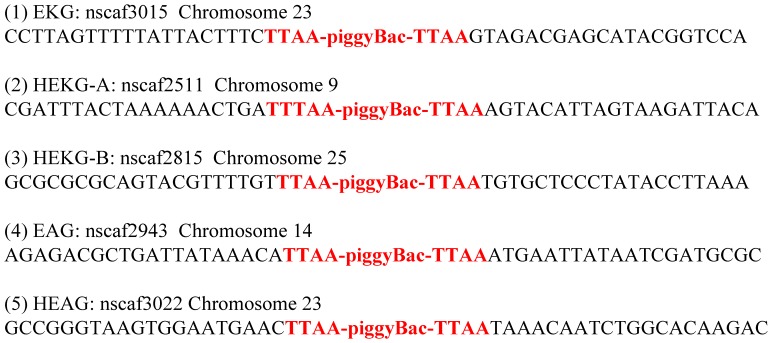
Analysis of the insertion sites of transgenic lines. The insertion sites were detected using inverse PCR. The genomic DNA from each line was digested fully using *Hae III*, and then self-ligated. Two pairs of transposon-specific primers pBacL and pBacR were used for the PCR amplification reaction. The PCR products were cloned and sequenced.

The genomic DNA in each transgenic line was extracted from G1 male moths. Inverse PCR was performed to detect the insertion sites in these transgenic lines. The left part of the insert and the next left part of the genome sequence of the insertion site were amplified using the transposon-specific primer pBacL, while the right part of the insert and the next right part of the genome sequence of the insertion site were amplified using the transposon-specific primer pBacR. PCR amplification using pBacL and pBacR showed that each transgenic line contained only one band (data not shown). This suggested that only a single copy was inserted into each transgenic line. These results were also confirmed by sequencing of the PCR-amplified products (Fig. 2). Bioinformatics analysis demonstrated that the insertion site in EKG was located in an intergenic region, while the nearest genes to the left and right of the insertion site were located within 11 kb and 50 kb. The insertion site of HEKG-A was also in an intergenic region, while the nearest genes to the left and right of the insertion site were within 96 kb and 248 kb. The insertion site of HEKG-B was in the BGIBMGA004763 gene intron, which was a predicted gene that with no Expressed Sequence Tags (ESTs) [33], [34], [35] and no similar gene in NCBI. Thus, the predicted BGIBMGA004763 gene was probably not a legitimate *B. mori* gene and the insert might not have affected the expression of normal genes. The insertion site of EAG was in an intergenic region, while the nearest genes to the left and right of the insertion site were within 3 kb and 39 kb. The insertion site of HEAG was located an intergenic region while the nearest genes to the left and right of the insertion site were within 10 kb and 13 kb.

### Expression Profile of Hycu-ep32 in Transgenic Lines

**Figure 3:**
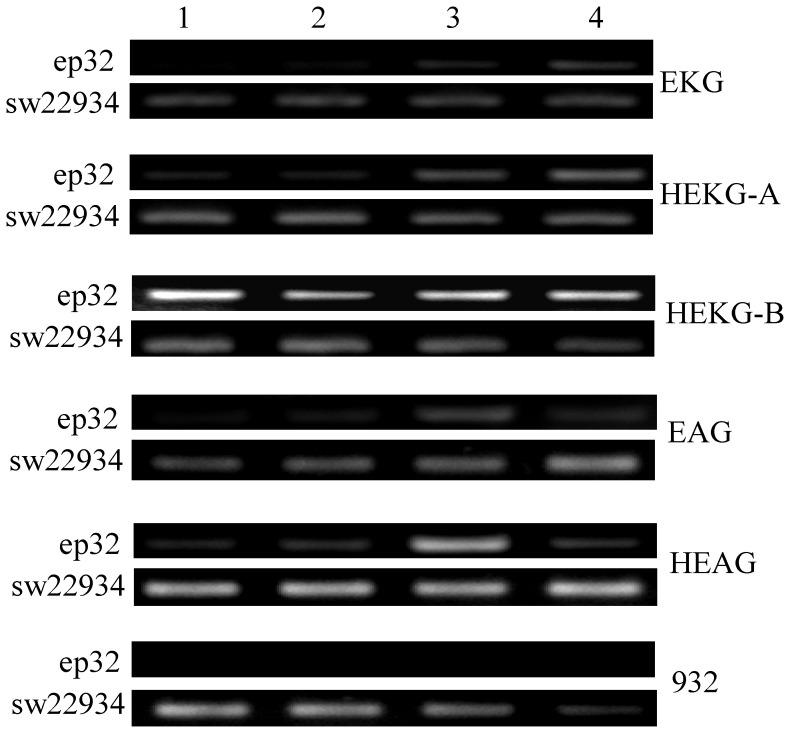
Analysis of *hycu-ep32* using RT-PCR in transgenic and non-transgenic silkworms. RNA was extracted from different developmental stages of EKG, HEKG-A, HEKG-B, EAG, HEAG, and 932, and then reverse-transcribed to cDNA. cDNA templates were used for RT-PCR. The PCR-amplified cycles of ep32 and sw22934 were 28 and 25, which detected the *hycu-ep32* gene and the silkworm house-keeping gene BGIBMGA003186, respectively. Points 1 to 4 represent third instar, third instar molt, fourth instar, and fourth instar molt larvae, respectively.

*hycu-ep32* has no homologous gene in the silkworm genome [33], [35]. To test whether *hycu-ep32* was expressed in each transgenic line, the cDNA templates of the third instar, third instar molt, fourth instar, and fourth instar molt larvae of EKG, HEKG-A, HEKG-B, EAG, HEAG, and 932 were used to detect *hycu-ep32* transcription. The PCR-amplified cycles of primers ep32QRT and sw22934 were 28 and 25, respectively. The RT-PCR results showed that *hycu-ep32* was expressed in most transgenic lines, but not in non-transgenic silkworm 932. The expression levels of *hycu-ep32* in the fourth instar were higher than those of the third instar in the transgenic lines (Fig. 3). There was almost no expression of *hycu-ep32* in the third instar and very low expression in the fourth instar in EKG. These results showed that 39 KP had little or no activity in silkworm larvae. The *hycu-ep32* expression level in HEKG was higher than that in EKG, which suggested that hr3 could enhance the activity of 39 KP in silkworm larvae. There were no differences in the *hycu-ep32* expression levels of EAG and HEAG. The *hycu-ep32* expression level in HEKG-B was the highest among all the lines tested. (Fig. 3). These results indicated that the exogenous *hycu-ep32* was successfully expressed in the transgenic lines.

### Determination of the Anti-BmNPV Capacity of Transgenic Lines

**Figure 4:**
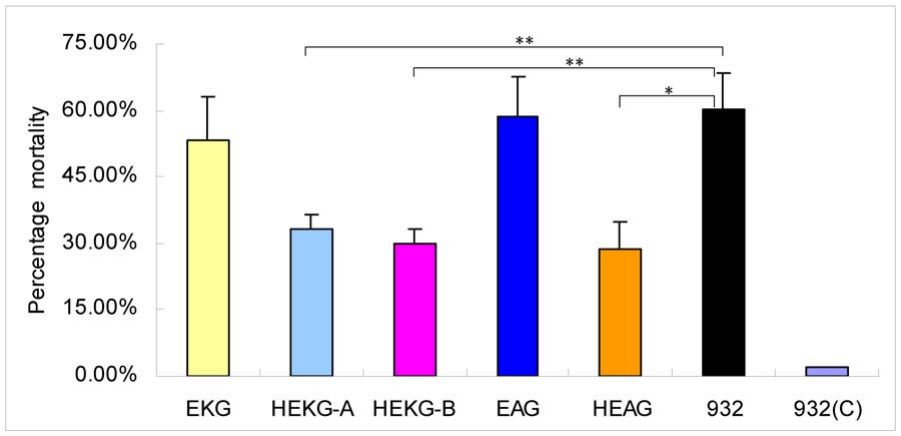
Mortality statistics after infection with BmNPV *per os* using third instar larvae. Six silkworm lines, i.e., EKG, HEKG-A, HEKG-B, EAG, HEAG, and 932, were infected orally with 3 × 10^5^ OB/larva using newly exuviated third instar larvae. The same quantity of OBs was applied to pieces of fresh mulberry leaves with a 1 cm diameter. Larvae that consumed an entire piece of leaf treated with OBs were selected for the subsequent rearing. The mortality of each line was the average of triplicate infection replicates. Each replicate consisted of 70 larvae. Mortality statistics were analyzed daily from the time of infection until 10 dpi. The accumulative mortality up to 10 dpi is shown for each line. 932(C) is the non-infected control. Bars represent the standard deviations. Statistically significant differences: * *P*<0.05, ** *P*<0.01.

To investigate the capacity of these transgenic lines to protect against BmNPV infection, the newly exuviated third instar larvae of EKG, HEKG-A, HEKG-B, EAG, HEAG, and 932 were orally inoculated with BmNPV using 3 × 10^5^ occlusion bodies (OB)/larva. The OBs were smeared on a piece of 1 cm diameter fresh mulberry leaf and any larvae that consumed an entire piece of OB-treated leaf were collected for continuous rearing in normal conditions. Each line was infected with BmNPV using three replicate groups, each of which contained 70 larvae. The mortality statistics were calculated each day until the 10th day postinfection (dpi). Compared with transgenic lines EKG and EAG, the transgenic lines HEKG-A, HEKG-B, and HEAG had a sustained and distinctive protective capacity (Fig. 4). The mortality of EKG, HEKG-A, HEKG-B, EAG, HEAG, and 932 were 53.08%, 33.11%, 30.00%, 58.42%, 28.64%, and 60.19%, respectively. There was little decrease in the mortality rate of EAG and EKG compared with 932. However, the mortality rates of HEKG-A, HEKG-B, and HEAG were decreased by 27.08%, 30.19%, and 31.55% compared with 932, respectively. Most untreated silkworms 932(C) survived (Fig. 4).

The second determination also used third instar larvae (Fig. S1). After BmNPV oral infection using 3 × 10^5^ OB/larva, the mortality of HEKG-A, HEKG-B, and HEAG was decreased by 37.53%, 37.31%, and 24.35% compared with 932, respectively. However, when the BmNPV infection dose was decreased to 2.5 × 10^5^ OB/larva, the mortality rate of EAG and EKG was not decreased compared with 932 (Fig. S1).

The third determination used fourth instar larvae (Fig. S2). The mortality rates of HEKG-A, HEKG-B, and HEAG were decreased by 12.80%, 21.66%, and 18.38% compared with 932, respectively, after infection with BmNPV *per os* using 10^6^ OB/larva. However, there were virtually no differences in the mortality rates of EKG, EAG, and 932 after infection using 8.3 × 10^5^ OB/larva (Fig. S2).

The mortality statistics indicated that the HEKG-A, HEKG-B, and HEAG lines had significantly higher resistance to BmNPV compared with EKG, EAG, and the non-transgenic silkworms. According to the three comprehensive tests of resistance analysis, the antivirus capacity of HEKG-B was the highest among all of the lines tested.

### Hycu-ep32 Expression after BmNPV Infection

**Figure 5:**
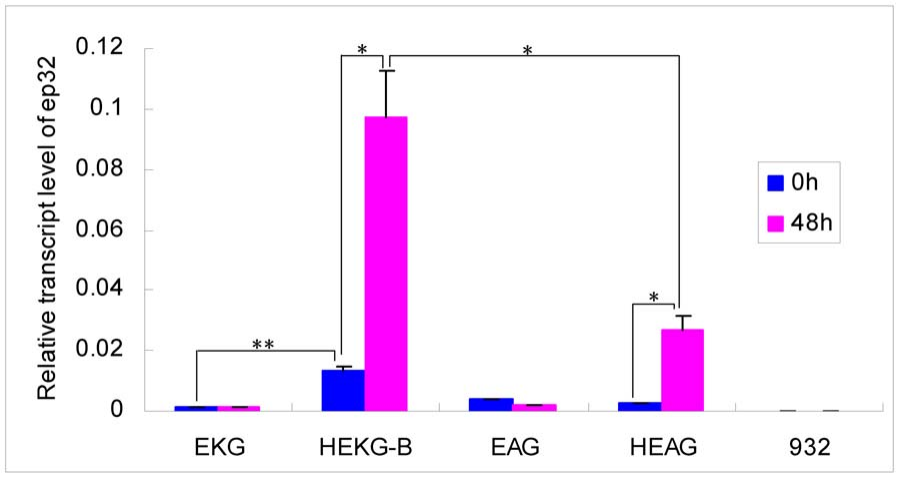
qPCR analysis of *hycu-ep32* expression after BmNPV infection of transgenic lines. The same viral quantity was inoculated into newly exuviated third instar larvae. The cDNA was extracted from EKG, HEKG-B, EAG, HEAG, and 932 using 10 treated larvae at 0 and 48 hpi, respectively. Primers ep32QRT were used for the qPCR analysis reactions. The time that the infection process ended was set as time point 0 h. Bars represent the standard deviations. Statistically significant differences: * *P*<0.05, ** *P*<0.01.

To confirm whether the expression of *hycu-ep32* was changed after BmNPV infection in the transgenic lines, the cDNA templates of EKG, HEKG-B, EAG, HEAG, and 932 at 0 and 48 h postinfection were used for qPCR analysis. qPCR showed that the expression levels of *hycu-ep32* in HEKG-B were the highest among all the lines, regardless of whether they were infected with BmNPV, whereas the expression levels of *hycu-ep32* in EKG were very low (Fig. 5). With a normal physical status, the expression level of *hycu-ep32* in HEKG-B was 11.92-fold that of EKG, while the expression level of *hycu-ep32* in HEAG was no higher than that of EAG. These results indicated that hr3 could significantly enhance the transcription capacity of 39 KP in normal silkworm larvae. At 48 hpi, the expression levels of *hycu-ep32* in EKG, HEKG-B, EAG, and HEAG were 1.32-fold, 7.41-fold, 0.52-fold, and 10.10-fold, respectively, compared with the insects before infection (Fig. 5). Thus, hr3 could significantly increase the transcription activity of 39 KP and A4P after BmNPV infection. After infection with BmNPV, the expression levels of antivirus factor *hycu-ep32* were induced effectively in HEAG, particularly in HEKG-B (Fig. 5), which suggested that hr3 combined with 39 KP was the best of all the promoters tested. These results also showed that the higher transcription of *hycu-ep32* mRNAs led to a higher anti-BmNPV capacity in the transgenic lines (Figs 4, S1, S2, 5).

### qPCR Analysis of Virus Proliferation after Inoculation

**Figure 6:**
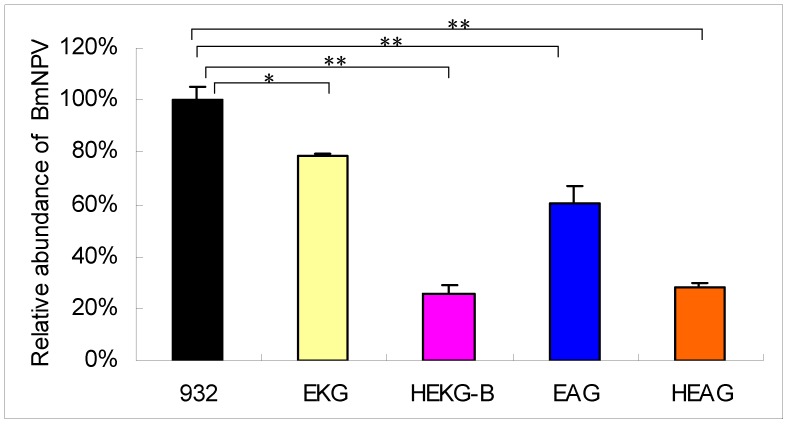
Analysis of BmNPV by qPCR in transgenic and non-transgenic lines after viral inoculation. The ingested viral dose of each individual was the same as that used for the newly exuviated third instar larvae. Total DNA was obtained from 10 treated larvae in each line at 48 hpi. The DNA-accumulated numbers of BmNPV were detected using qPCR with GP41 primers. The average of non-transgenic 932 was set at 100% and the values for transgenic lines were normalized against that of 932. Bars represent the standard deviations. Statistically significant differences: * *P*<0.05, ** *P*<0.01.

To further detect the antiviral capacity of each transgenic line against BmNPV, the number of BmNPV in the bodies of transgenic and non-transgenic silkworm lines were analyzed after infection. The viral dose ingested by each individual was the same as that used at the stage of newly exuviated third instar larvae. Total DNA was extracted from 10 treated larvae in each line at 48 hpi. The DNA-accumulated virus number was determined via qPCR using GP41 primers [10]. The virus content of the non-transgenic 932 was set to 100% and the values of the transgenic lines were normalized against this value. Thus, the levels of BmNPV DNA accumulated in EKG, HEKG-B, EAG, and HEAG were 78.43%, 25.48%, 60.72%, and 28.39%, respectively (Fig. 6). The virus content of all the transgenic lines was lower than that of the control at 2 dpi, particularly in the transgenic lines HEKG-B and HEAG. These results suggested that transgenic lines could inhibit BmNPV proliferation.

### Economic Characteristic of the Transgenic Lines

**Figure 7:**
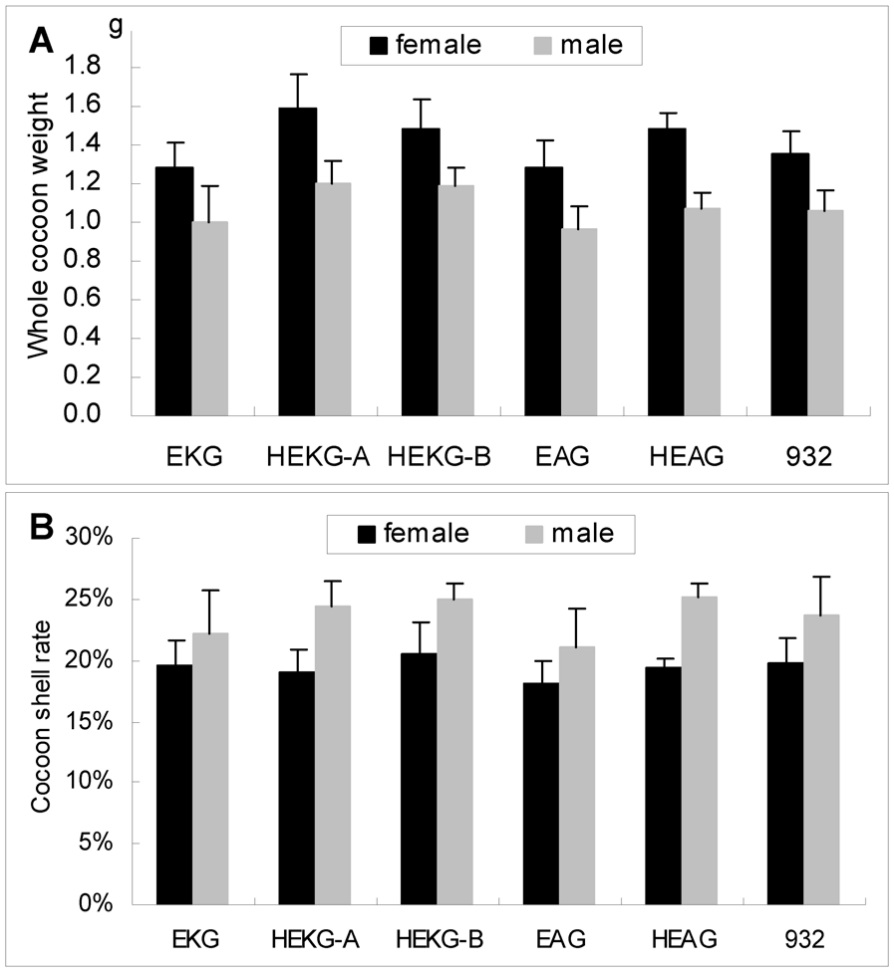
Economic characteristics analysis. The economic characteristics of EKG, HEKG-A, HEKG-B, EAG, HEAG, and 932 were investigated. Fifteen cocoons from male individuals and 15 cocoons from female individuals were selected randomly from each line. The weights of the entire cocoon and the cocoon shell were determined for each individual. The cocoon shell rate was the ratio of the cocoon shell weight to the overall cocoon weight. Each value was the average of 15 replicate tests. Bars represent the standard deviations.

The sericulture industry is very important for silk production, but it faces great challenges because of severe diseases. Traditional methods of breeding resistant silkworm varieties might enhance their antivirus capacity but reduce their economic characteristics. To investigate the effects of *hycu-ep32* overexpression in transgenic lines on their economic characteristics, we investigated the overall cocoon weights and cocoon shell rates of the transgenic lines. The cocoon shell rate is the ratio of the cocoon shell weight to the overall whole cocoon weight. There were no obvious differences in the economic characteristics of the transgenic and non-transgenic lines (Fig. 7). This showed that *hycu-ep32* overexpression did not affect the economic characteristics of transgenic silkworms.

## Discussion

*B. mori* is a typical lepidopteran model and it is economically important in the silk industry [33], [34], [35]. However, BmNPV causes severe annual economic losses in the sericulture industry. In our previous study, we reported that transgenic silkworms had significantly improved anti-BmNPV capacity due to overexpression of the endogenous antiviral gene *Bmlipase-1* [10]. In the current study, we further optimized the method of vector construction and induced the overexpression of an exogenous antiviral gene, before analyzing its anti-BmNPV capacity.

The *hycu-ep32* gene of HycuNPV can restrict BmNPV proliferation in co-infected cells [30]. In this study, we constructed four overexpression vectors where *hycu-ep32* was driven by 39 KP, hr3+39 KP, A4P, or hr3+A4P. The corresponding transgenic lines EKG, HEKG-A and HEKG-B, EAG, HEAG were generated to test their resistance. As expected, the expression level of exogenous *hycu-ep32* was increased significantly after infection with BmNPV in HEKG-B, which had a significantly enhanced anti-BmNPV capacity. Importantly, the economic characteristics of the transgenic strains remained the same.

*Hycu-ep32* was induced abundantly after BmNPV infection of HEKG-B and HEAG (Fig. 5) where hr3 was upstream of 39KP and A4P, which controlled the expression of *hycu-ep32* (Fig. 1). It was assumed that hr3 was transactivated by the IE1 protein that was produced by the virus after BmNPV infection. The expression level of *hycu-ep32* in HEKG-B (Fig. 5) and the resistance of HEKG-B (Figs 4, S1, S2) were the highest among all the detected transgenic lines, suggesting that hr3 combined with 39KP gave the best performance of all the promoters tested. Compared with target genes expressed stably by using constitutive promoters [8], [9], [11], [12], the expression of *hycu-ep32* was appropriate with normal physical status, while it increased significantly as the virus content of HEKG-B increased.

The 39KP activity was very low in EKG (Figs 3, 5), which was only slightly enhanced after BmNPV infection (Fig. 5). However, the 39 K promoter of AcMNPV had no activity, although it could be activated by the virus in cells [17]. This difference may be attributable to differences in BmNPV and AcMNPV, or different individual larvae and cell lines. The transgenic lines EKG and EAG inhibited the multiplication of BmNPV to some extent (Fig. 6) via the expression of *hycu-ep32* (Figs 3, 5). However, the expression of *hycu-ep32* was not sufficiently high to decrease the mortality rate (Figs 4, 5, S1, S2). The anti-BmNPV capacity was enhanced significantly due to the high level expression of *hycu-ep32* after infection in HEKG-B. Hycu-ep32 can induce a severe global protein synthesis shutdown, thereby suppressing BmNPV multiplication in co-infected cells [30]. We assumed that hycu-ep32 could suppress BmNPV DNA replication or protein synthesis in transgenic larvae.

The overexpression of anti-pathogen genes or downregulation of pathogen genes by transgenic RNAi in hosts are proven and effective disease-control strategies. However, there have been no previous reports of enhanced disease resistance in animals due to the overexpression of exogenous anti-pathogen genes. Resistance to BmNPV can be boosted by overexpressing endogenous *Bmlipase-1* in transgenic silkworm [10] or knocking down the *ie-1* gene using transgenic RNAi [7]. A combination of these methods would be more effective. In previous reports, transgenic silkworms were created using nondiapause or non-practical silkworm [6], [7], [10], [31], [32]. In the current study, we generated the first transgenic silkworm using a practical diapause strain.

In conclusion, we successfully generated transgenic practical silkworms with an exogenous antiviral gene via embryo microinjection. The expression level of the antiviral factor was significantly upregulated with an increase in the viral content after infection. The transgenic silkworms had an enhanced anti-BmNPV capacity with no negative effects on their economic characteristics, which means they could be applied in sericulture to enhance the resistance of silkworms. This is the first report of disease resistance via overexpression of an anti-pathogen factor using an inducible promoter and enhancer in animals, and it may pave the way for disease control studies in other organisms in the future.

## Materials and Methods

### Silkworm Strain and Virus

The practical diapause silkworm strain “932” was maintained at the Gene Resource Library of Domesticated Silkworm (Southwest University, Chongqing, China). The silkworm was reared on fresh mulberry leaves under standard conditions. The larvae underwent oral inoculation with wild BmNPV (Guangdong strain, China) and the OBs were harvested from larvae hemolymph before the larvae died.

### Transgenic Vectors Construction

DNA was extracted from HycuNPV OBs and BmNPV OBs using a MiniBEST viral RNA/DNA Extraction Kit Ver. 3.0 (TaKaRa). The *hycu-ep32* gene was amplified from the HycuNPV genomic DNA using the primers ep32F (5′-CGGGATCCATGAAGAACCAACAACAG-3′) and ep32R (5′-ATAGTTTAGCGGCCGCTTAATTTATTAACATATCAAAG-3′) [30]. The promoter sequence of the 39K gene (39KP) was amplified from the BmNPV genomic DNA using the primers 39KPF (5′-ACGCGTCGACCTTGACCCGAAGCGAAAT-3′) and 39KPR (5′-CGCGGATCCTGTTGCTCCGGCATGTTT-3′) (provisional Chinese Patent No. 201010231957.9, Pan *et al*.). The BmNPV hr3 enhancer (provisional Chinese Patent No. 201110423280.3, Xia *et al*.), *B. mori* A4 promoter (A4P), and termination signal SV40 were preserved in the laboratory. The 39KP, *hycu-ep32*, and SV40 were added to *piggyBac* [3×p3 EGFP afm] [31], [32] to generate transgenic vector *piggyBac* [39KP-ep32-SV40-3×p3 EGFP afm] (abbreviated as pb-EKG). hr3 was added to pb-EKG to generate the transgenic vector pb-HEKG. A4P, *hycu-ep32*, and SV40 were added to *piggyBac* [3×p3 EGFP afm] to generate the transgenic vector *piggyBac* [A4P-ep32-SV40-3×p3 EGFP afm] (abbreviated as pb-EAG). hr3 was added to pb-EAG to generate the transgenic vector pb-HEAG (Fig. 1).

### Microinjection and Screening

The practical silkworm strain 932 was used for the transgenic embryo microinjection, which is a diapause strain. The 932 embryos were incubated at 15°C after acidic treatment and the larvae were fed with mulberry leaves under standard conditions [10]. After this processes, most of the embryos were nondiapaused in the next generation. Mixtures of transgenic vector (400 ng/µL) and helper plasmid pHA3PIG (400 ng/µL) were injected into the nondiapause 932 embryos within 2 h of oviposition [10], [31], [32]. The G1 embryos were screened for EGFP protein in the ocelli using a fluorescent microscope (Olympus) [10], [32]. EGFP-positive moths were then sibling-mated to generate offspring in each G1 brood for each transgenic vector. One transgenic line of pb-EKG (known as EKG), two transgenic lines of pb-HEKG (known as named HEKG-A and HEKG-B), one transgenic line of pb-EAG (known as EAG), and one transgenic line of pb-HEAG (known as HEAG) were screened.

### Identifying the Insertion Site

Genomic DNA was extracted from EKG, HEKG-A, HEKG-B, EAG and HEAG in G1 male moths. There was a *Hae III* site in the left terminal inverted repeats (PL) and right terminal inverted repeats (PR) of *piggyBac* [3×p3 EGFP afm] vector, respectively. About 20 µg of the genomic DNA from each sample was fully digested using *Hae III* for 10 h at 37°C, then purified and self-ligated with Solution I (NEB) [10]. Each ligated product was PCR-amplified using the transposon-specific primers pBacL and pBacR (pBacL F: 5′-ATCAGTGACACTTACCGCATTGACA-3′, pBacL R: 5′-TGACGAGCTTGTTGGTGAGGATTCT-3′; pBacR F: 5′-TACGCATGATTATCTTTAACGTA-3′, pBacR R: 5′- GTACTGTCATCTGATGTACCAGG-3′), respectively. The PCR products were cloned and sequenced.

### Analysis of the Expression Level of Hycu-ep32 by RT-PCR

Different developmental stages of EKG, HEKG-A, HEKG-B, EAG, HEAG, and non-transgenic 932 were used for RNA extraction. The total RNA was extracted using a total RNA (*mini*) kit (Watson) and digested with 20 U Rnase-free Dnase I (Promega). Approximately 4 µg of treated RNA was reverse-transcribed in a 25 µL reaction system using M-MLV reverse transcriptase (Promega). Each template was diluted with 100 µL, before 1 µL of cDNA was used for RT-PCR reactions using the primers ep32QRT (F: 5′-ACATCAGAATACCCATCACG-3′, R: 5′-ATTGTTCAATGGTAACTCCC-3′). The primers sw22934 for the housekeeping gene BGIBMGA003186 (F: 5′-TTCGTACTGGCTCTTCTCGT-3′, R: 5′-CAAAGTTGATAGCAATTCCCT-3′) were used as the controls.

### Mortality Analysis

The mortality of different transgenic strains and non-transgenic strain were investigated after being infected with wild BmNPV *per os*. The infection testing of each line used three replicates and each replicate was performed with 70 larvae. Fresh mulberry leaves were cut into 1 cm or 1.5 cm diameter round pieces and then treated with solution containing OBs. Newly exuviated 3 third instar or fourth instar larvae were used in the test. Individuals in the one-repeat experiment were confirmed as consuming equal quantities of OBs by feeding them individually [10]. There were three replicates of non-infection and every replicate consisted of 70 larvae. The mortality rate was analyzed from the time of infection until 10 dpi.

### Confirmation of the Change in Hycu-ep32 after Virus Induction by qPCR

Total RNA was extracted from EKG, HEKG-B, EAG, HEAG, and 932 using 10 treated larvae at 0 and 48 hpi. The time that the infection process ended was set as time point 0 h. cDNA templates from 10 biological materials were used for qPCR reactions with ep32QRT primers on the ABI StepOnePlus™ Real-Time PCR System (Applied Biosystems). BGIBMGA003186 was used as a reference gene to standardize the variance of the different templates. Each assay was performed three times.

### qPCR Analysis of Virus after BmNPV Infection

Total DNA was obtained from the larvae of EKG, HEKG-B, EAG, HEAG, and 932 at 48 hpi. Each sample that was extracted from 10 treated larvae was used as templates for qPCR. The DNA templates (20 ng) were PCR-amplified using primers for the BmNPV *GP41* gene (F: 5′-CGTAGTAGTAGTAATCGCCGC-3′, R: 5′-AGTCGAGTCGCGTCGCTTT-3′) [10], [36] using an ABI StepOnePlus™ Real-Time PCR System. *BmGAPDH* (F: 5′-CATTCCGCGTCCCTGTTGCTAAT-3′, R: 5′-GCTGCCTCCTTGACCTTTTGC-3′) was used as an internal control to standardize the variance of the different templates [10], [36]. The test was performed three times.

### Comparison of the Economic Characteristics

*B. mori* is an important insect for the commercial production of silk. The economic characteristics of EKG, HEKG-A, HEKG-B, EAG, HEAG, and 932 were investigated to determine whether *hycu-ep32* overexpression might affect the silk production of transgenic lines. Fifteen cocoons from male individuals and 15 cocoons from female individuals were selected randomly from each line for analysis. The weights of the entire cocoon and the cocoon shell were determined for each individual.

## Supporting Information

Figure S1 **The second time of resistance detection using third instar larvae.** EKG, EAG, and 932 (white) were infected orally with 2.5 × 10^5^ OB/larva using newly exuviated third instar larvae. HEKG-A, HEKG-B, HEAG, and 932 (black) were infected with 3 × 10^5^ OB/larva *per os* at third instar larvae. The ingested viral dose of each individual in each replicate was the same. The mortality of each line was the average of triplicate infection replicates. Each replicate consisted of 70 larvae. Mortality statistics were analyzed daily from the time of infection until 10 dpi. The accumulative mortality up to 10 dpi is shown for each line. 932(C) is the non-infected control. Bars represent the standard deviations. Statistically significant differences: * *P*<0.05, ** *P*<0.01.
(TIF)

Figure S2 **Mortality statistics after infection with BmNPV** ***per os*** **using fourth instar larvae.** The solution of OBs was smeared on pieces of fresh mulberry leaf with 1.5 cm diameter. EKG, EAG, and 932 (white) were infected orally with 8.3 × 10^5^ OB/larva using newly exuviated fourth instar larvae. HEKG-A, HEKG-B, HEAG, and 932 (black) were infected with 10^6^ OB/larva *per os* at fourth instar larvae. The ingested viral dose of each individual in each replicate was the same. The mortality of each line was the average of triplicate infection replicates. Each replicate consisted of 70 larvae. Mortality statistics were analyzed daily from the time of infection until 10 dpi. The accumulative mortality up to 10 dpi is shown for each line. 932(C) is the non-infected control. Bars represent the standard deviations. Statistically significant differences: * *P*<0.05, ** *P*<0.01.
(TIF)
