# Supplementary figures and images for: Resistance to BmNPV via Overexpression of an Exogenous Gene Controlled by an Inducible Promoter and Enhancer in Transgenic Silkworm, Bombyx mori
Source: PLoS One. 2012 Aug 1;7(8):e41838. doi: 10.1371/journal.pone.0041838 (PMC3411602; doi:10.1371/journal.pone.0041838)

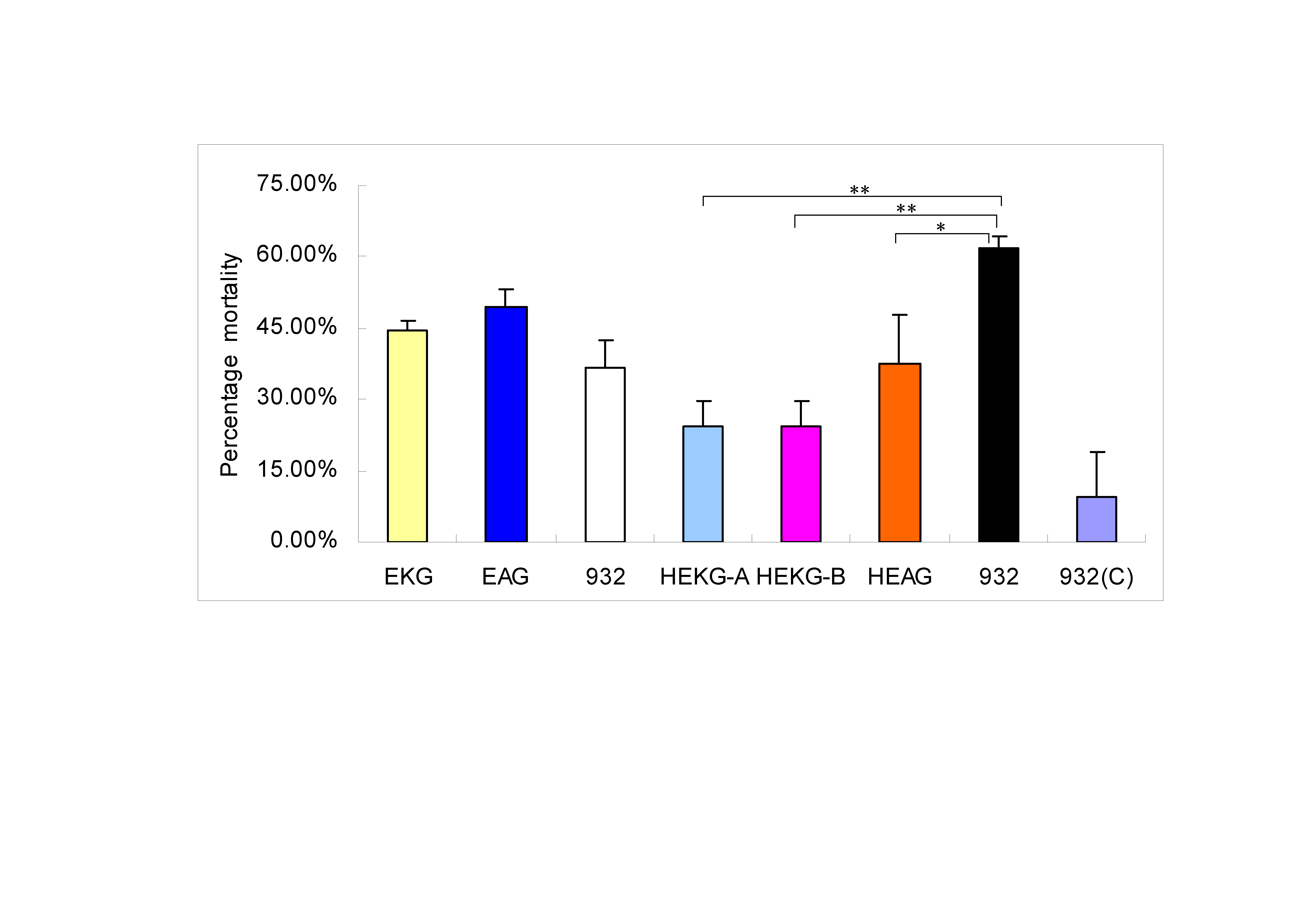

Supplement: Figure S1 — The second time of resistance detection using third instar larvae. EKG, EAG, and 932 (white) were infected orally with 2.5 × 10^5^ OB/larva using newly exuviated third instar larvae. HEKG-A, HEKG-B, HEAG, and 932 (black) were infected with 3 × 10^5^ OB/larva per os at third instar larvae. The ingested viral dose of each individual in each replicate was the same. The mortality of each line was the average of triplicate infection replicates. Each replicate consisted of 70 larvae. Mortality statistics were analyzed daily from the time of infection until 10 dpi. The accumulative mortality up to 10 dpi is shown for each line. 932(C) is the non-infected control. Bars represent the standard deviations. Statistically significant differences: * P<0.05, ** P<0.01. (TIF) [file pone.0041838.s001.tif]

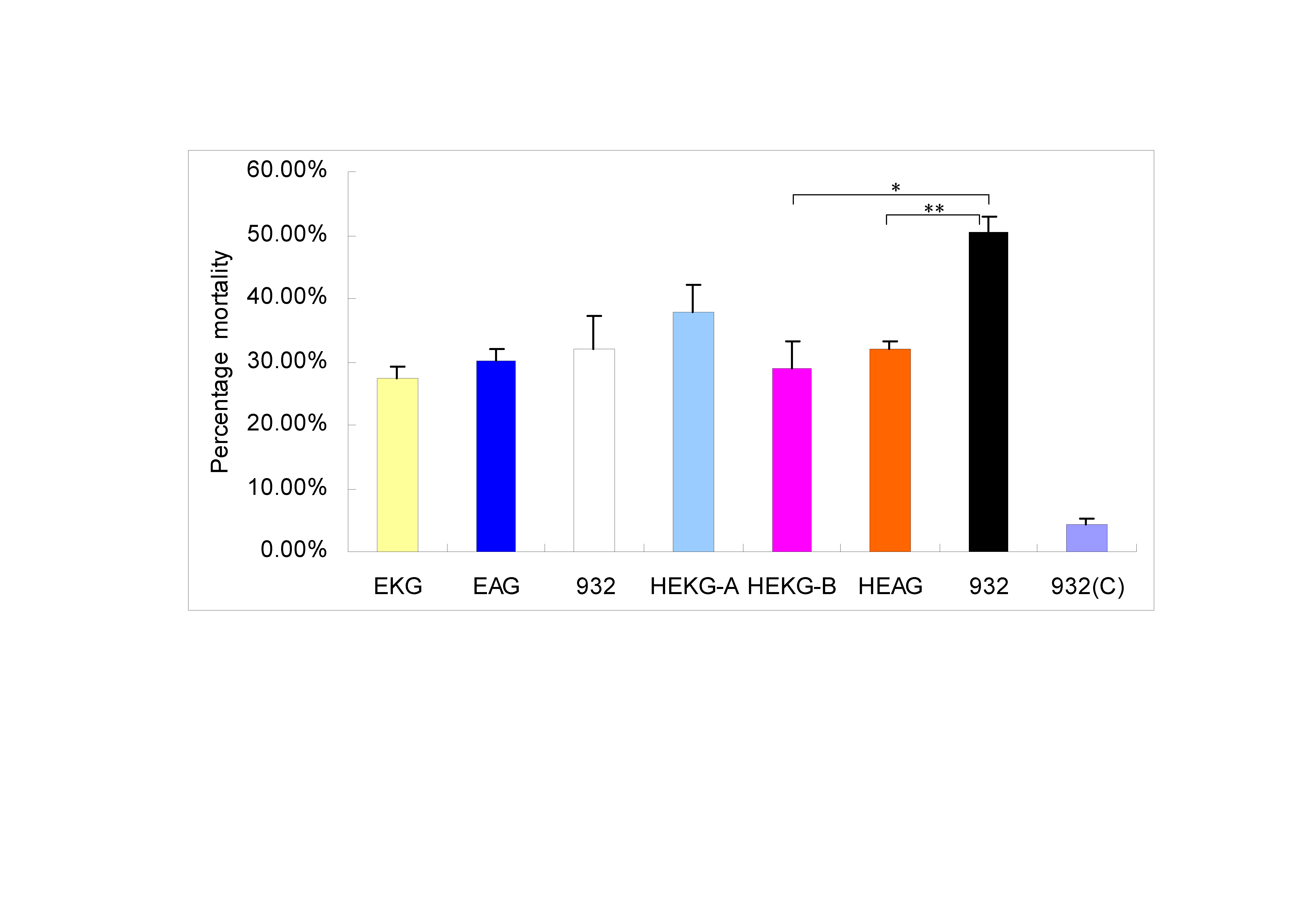

Supplement: Figure S2 — Mortality statistics after infection with BmNPV per os using fourth instar larvae. The solution of OBs was smeared on pieces of fresh mulberry leaf with 1.5 cm diameter. EKG, EAG, and 932 (white) were infected orally with 8.3 × 10^5^ OB/larva using newly exuviated fourth instar larvae. HEKG-A, HEKG-B, HEAG, and 932 (black) were infected with 106 OB/larva per os at fourth instar larvae. The ingested viral dose of each individual in each replicate was the same. The mortality of each line was the average of triplicate infection replicates. Each replicate consisted of 70 larvae. Mortality statistics were analyzed daily from the time of infection until 10 dpi. The accumulative mortality up to 10 dpi is shown for each line. 932(C) is the non-infected control. Bars represent the standard deviations. Statistically significant differences: * P<0.05, ** P<0.01. (TIF) [file pone.0041838.s002.tif]
